# Building capacity for antiretroviral delivery in South Africa: A qualitative evaluation of the PALSA PLUS nurse training programme

**DOI:** 10.1186/1472-6963-8-240

**Published:** 2008-11-18

**Authors:** J Stein, S Lewin, L Fairall, P Mayers, R English, A Bheekie, E Bateman, M Zwarenstein

**Affiliations:** 1 University of Cape Town Lung Institute, George St, Mowbray 7700, Cape Town, South Africa; 2 Department of Public Health and Policy, London School of Hygiene and Tropical Medicine, UK; 3 Health Systems Research Unit, Medical Research Council of South Africa, PO Box 19070, Tygerberg 7505, Cape Town, South Africa; 4 University of Cape Town Lung Institute, George St, Mowbray 7700, Cape Town, South Africa; 5 School of Health and Rehabilitation Sciences, Faculty of Health Sciences, University of Cape Town, South Africa; 6 University of Cape Town Lung Institute, George St, Mowbray 7700, Cape Town, South Africa; 7 University of Cape Town Lung Institute, George St, Mowbray 7700, Cape Town, South Africa; 8 School of Pharmacy, University of the Western Cape, P/Bag X17, Bellville 7535, Cape Town, South Africa; 9 Department of Medicine, University of Cape Town Lung Institute, George St, Mowbray, Cape Town, South Africa; 10 Centre for Health Services Sciences, Sunnybrook Research Institute, Toronto, Ontario, Canada; 11 Department of Health Policy, Management and Evaluation, University of Toronto, Toronto, Ontario, Canada

## Abstract

**Background:**

South Africa recently launched a national antiretroviral treatment programme. This has created an urgent need for nurse-training in antiretroviral treatment (ART) delivery. The PALSA PLUS programme provides guidelines and training for primary health care (PHC) nurses in the management of adult lung diseases and HIV/AIDS, including ART. A process evaluation was undertaken to document the training, explore perceptions regarding the value of the training, and compare the PALSA PLUS training approach (used at intervention sites) with the provincial training model. The evaluation was conducted alongside a randomized controlled trial measuring the effects of the PALSA PLUS nurse-training (Trial reference number ISRCTN24820584).

**Methods:**

Qualitative methods were utilized, including participant observation of training sessions, focus group discussions and interviews. Data were analyzed thematically.

**Results:**

Nurse uptake of PALSA PLUS training, with regard not only to ART specific components but also lung health, was high. The ongoing on-site training of all PHC nurses, as opposed to the once-off centralized training provided for ART nurses only at non-intervention clinics, enhanced nurses' experience of support for their work by allowing, not only for ongoing experiential learning, supervision and emotional support, but also for the ongoing managerial review of all those infrastructural and system-level changes required to facilitate health provider behaviour change and guideline implementation. The training of all PHC nurses in PALSA PLUS guideline use, as opposed to ART nurses only, was also perceived to better facilitate the integration of AIDS care within the clinic context.

**Conclusion:**

PALSA PLUS training successfully engaged all PHC nurses in a comprehensive approach to a range of illnesses affecting both HIV positive and negative patients. PHC nurse-training for integrated systems-based interventions should be prioritized on the ART funding agenda. Training for individual provider behaviour change is nonetheless only one aspect of the ongoing system-wide interventions required to effect lasting improvements in patient care in the context of an over-burdened and under-resourced PHC system.

## Background

In South Africa, where over 5 million people are HIV infected, and at least 900 000 people are estimated to be eligible for antiretroviral treatment (ART), the roll-out of the ART programme thus far has been slow and insufficient [1]. Nonetheless, it is estimated that more than 300 000 people have now accessed ART in South Africa through the public sector [2]. The rapid expansion of the ART programme has refocused attention on the need for the provision of *integrated *primary health care. This is partly because HIV, with its diverse manifestations, demands it. It is also because without an integrated health systems approach, the South African ART programme, heavily-injected with donor funding and potentially the largest in the world, would become a mammoth vertical structure of little benefit to the overall health service [3-5]; which is fragile, overburdened and in urgent need of additional resources. The pool of human resources in particular is so low that a vertical ART programme would continue, as has already been seen, to drain experienced personnel from the wider health service. The most compelling reason for an integrated PHC approach is that PHC in South Africa is conducted, almost exclusively, by nurses. It is the only level of the public health care structure which reaches most South African people, and therefore the only way for the ART programme to reach all those who need it. In the face of one of the most extensive AIDS epidemics in the world and a critical shortage of health care workers, especially doctors [6-8], it is clear that non-physician models of health care delivery are necessary if the constantly expanding ART programme is to be successful and sustainable [9].

### ART roll-out in the Free State Province

The Free State province of South Africa has a high prevalence of HIV infection, with 31.1% of antenatal attendees testing positive [10]. Demographic modeling suggests that 48 392 of the Free States 391 527 HIV-positive citizens have AIDS [11], and so are presently eligible for ART in line with the National AIDS Plan [12]. Roll-out of the Free State ART programme started in May 2004 and at the end of March 2005, only 1 165 had commenced treatment [13]. These figures have since increased and 9320 patients had commenced treatment by July 2007 (Venessa Timmerman, personal communication, as per FSDoH data warehouse, 2007).

The Free State, like South Africa as a whole, is characterized by a lack of skilled health workers. This is illustrated by the vacancy rate in 2005 of 40.7% for health professional posts in the province – a figure substantially higher than the national average of 31.1%. [14]. PHC nurses play a pivotal role in the ART programme [15,16]. The roles and responsibilities of PHC nurses in the Free State ART programme have already grown substantially since the time of this study. At that time, which co-incided with the introduction of the programme in selected sites, nurses were responsible for screening and diagnosing patients; and assessing and referring those who qualified for treatment (i.e. those people with a CD4 count = 200 and/or AIDS as diagnosed by clinical staging) to the nearest doctor (usually at a hospital) for assessment. Thereafter patients were referred back to nurse-run clinics where nurses issued medication and conducted clinical monitoring. A doctor reviewed progress twice during the first three months, and thereafter six-monthly.

### The PALSA PLUS approach

PALSA PLUS is a health systems-based approach to training for primary care providers which attempts to capitalize on the opportunity provided by the ART roll-out to ensure that training for the ART programme is also used to strengthen overall health service delivery. This is in response to concerns that global initiatives such as '3 by 5' [1] have emphasized increasing the number of patients on ART without giving sufficient consideration to impacts on the primary health system [3,4].

PALSA PLUS is based on the PALSA (Practical Approach to Lung Health in South Africa) programme. PALSA has been shown in a randomized controlled trial (RCT) to be a promising model for improving quality of care and the control of priority respiratory diseases, without extra staff, in resource poor primary care settings [17]. PALSA PLUS was developed at the request of the Free State province following the successful implementation of PALSA.

The "+" in PALSA PLUS was added when the programme was adapted and reconceptualized to incorporate the management of HIV and AIDS, including ART. The inclusion in PALSA PLUS of HIV/AIDS management is based on the rationale that South Africa has the highest co-infection rate of HIV and TB in the world [18], and that TB is the leading cause of death among HIV infected South Africans [19]. It therefore made clinical sense to combine a lung health training programme with the additional training on ART now required. This approach was seen as having the added advantage of protecting against vertical ART programme dominance and retaining attention on the management of under-diagnosed and under-treated priority diseases, including those that are HIV-related, such as tuberculosis, and other illnesses, such as asthma [5,17].

### The PALSA PLUS guideline and training programme

The PALSA PLUS intervention included two main components: (1) a comprehensive set of algorithm-based syndromic guidelines for the PHC nurse clinical management of respiratory diseases and HIV/AIDS; and (2) a training programme to facilitate guideline implementation. While the PALSA PLUS guidelines were evidence-based, they were also tailored to existing national and provincial models of care and designed to be applicable in under-resourced PHC clinical settings. The guidelines were designed to be as simple and self-explanatory as possible. The intervention nonetheless involved a training programme to facilitate guideline use, given that the impacts of passive guideline dissemination on practice are modest [20].

PALSA PLUS nurse-training was based on the principles of educational outreach – a strategy shown to be effective in changing private health provider behaviour and practice [21]. These principles include the provision of short, face-to-face, in-service, interactive training by a trusted outsider. In accordance with educational outreach strategy, both the guidelines themselves and the training programme emphasized specific key behaviour change messages (i.e., relevant evidence-based diagnostic or treatment messages) which were seen to be most strongly correlated with improved treatment outcomes [22,23].

Using a 'cascade' training approach [24], nurse-trainers were trained in both the content of the guideline and learner-centered group facilitation in order to transfer relevant knowledge and skills to PHC nurses at clinic level. Basic counselling principles, emphasizing the importance of guiding the client in taking responsibility for all health-related decision-making, were also conveyed and practiced in role-plays focusing on common clinical encounters. The core development team for the guidelines and for the training of the nurse-trainers included three medical doctors specializing in TB and AIDS care and a training facilitator with extensive expertise and experience in the nursing sector. Nine nurse-trainers, with varying degrees of medical knowledge and prior nurse-training experience and already employed by the provincial Department of Health as TB and HIV co-coordinators, were trained in a five day intensive live-in course. This training was followed by three six-weekly support visits from the core guideline and training development team during the course of each nurse-trainer's provision of training to PHC nurses at clinics province-wide. Informal individual support contacts with nurse-trainers were also provided.

The on-site training of PHC clinic nurses by nurse-trainers was conducted in weekly two hour sessions over a period of three to four months. This is in accordance with evidence to the effect that the alternation of learning and practice is the preferential model for the integration, testing and application of new knowledge [25,26].

In order to help ensure replicability across the province, the duration and quality of both the training for nurse-trainers and the on-site training of nurses themselves, was determined wholly by the constraints, particularly with regard to human resources, of the provincial Department of Health.

### Intervention and research design

The fifteen clinics chosen by the Free State Department of Health to be involved in the first phase of the provincial ART roll-out were randomized to receive either the PALSA PLUS guidelines and a one week provincial training course only (control sites, n = 7) or to receive the provincial training and PALSA PLUS guidelines *as well as *an additional PALSA PLUS training package (intervention sites, n = 8) (Table 1).

**Table 1:** Interventions received by the control and intervention sites

**Control sites (n = 7)**	**Intervention sites (n = 8)**
Provincial training (ART nurses only)	Provincial training (ART nurses only)
Dissemination of PALSA PLUS guidelines to ART nurses only	Dissemination of PALSA PLUS guidelines **and **PALSA PLUS educational outreach training to *all* clinic nurses

The provincial training was provided to selected nurses newly appointed to the ART treatment programme (so-called "ART nurses") at both intervention and control sites and was conducted prior to the initiation of PALSA PLUS training at intervention sites. It is important to note that only the one or two nurses designated as "ART nurses" in each PHC clinic attended this provincial training. Provincial training included an introduction to the PALSA PLUS guideline and all nurses were given a copy of the guideline in their resource packs. Nurses were also provided with a structured opportunity to practice using the guidelines in role-plays. In all other respects, the provincial training may be described as essentially didactic in style, involving unidirectional presentations and passive dissemination of information and resource materials.

Thereafter, eight intervention sites received additional on-site PALSA PLUS training. The current evaluation compares and contrasts clinics which received the provincial training only with those that also received on-site, interactive training in the use of the PALSA PLUS guidelines. Table 2 highlights the key differences between the provincial and PALSA PLUS training approaches.

**Table 2:** Comparison of provincial and PALSA PLUS training approaches

**Provincial training approach**	**PALSA PLUS training approach**
Centralized by district	Decentralized (on-site at individual clinics)
Doctors and nurses trained together	Training designed specifically for nurses
Attendance by ART nurses only	Attendance by all nurses in each intervention clinic
Conference-type format: Expert presentations (primarily didactic and uni-directional) by multiple specialists	Educational outreach (facilitative, interactive) by trained group facilitators
Predominantly ART-specific AIDS care	Lung health (including TB), HIV/AIDS (including ART) care

Multiple materials, including the PALSA PLUS guideline	PALSA PLUS guideline (and support materials) only

### Research Aim and Objectives

The qualitative evaluation elaborated here explored the perceptions of those involved in the ART programme, especially PHC nurses, regarding the value of the PALSA PLUS training approach. This evaluation was grounded within a larger contextual exploration of nurse perceptions of the Free State ART roll-out, the results of which are discussed elsewhere [15]. The evaluation was conducted alongside an randomized trial measuring patient outcomes attendant on PALSA PLUS nurse-training, the results of which are forthcoming (Trial reference number ISRCTN24820584).

## Methods

Qualitative data was collected in 2004 and 2005 using a variety of methods, including:

• Unstructured participant observation of the full five-day provincial training programme for 'ART nurses', i.e., those nurses selected to be involved in ART provision;

• Unstructured participant observation of the full five-day PALSA PLUS training of nurse-trainers and of all follow-up support workshops for nurse-trainers;

• Focus group discussions (n = 3) with all nine nurse-trainers during the course of implementation of the training;

• Participant observation (n = 3 sessions) of on-site PALSA PLUS nurse-training;

• Semi-structured interviews (n = 14) with four doctors in charge of district-level HIV clinics and ten nurses at two intervention and two control clinics.

Participant observation of provincial and PALSA PLUS training allowed for comparison of the training approaches adopted and nurses' responses to these. Both interviews and focus group discussions focused on respondents' perceptions of the value of the training they had received and their remaining training and support needs.

All participant observation and interview data were collected in English which was the language in which training was conducted. All data were tape-recorded, transcribed verbatim and analysed thematically. For each key analytic theme, data extracts were identified on the basis of being representative and/or interesting illustrations of an emerging issue [27]. All negative instances of the findings were discussed and accounted for.

The reliability and validity of the data were enhanced through iterative data collection, the use of a multi-method design and the ongoing discussion of findings within the research team for scrutiny and feedback [28,29]. Purposive sampling ensured that those clinics chosen for detailed study included both urban and rural sites with varying degrees of human and financial resources [30].

The study on which this paper is based has full ethical approval from the Research Ethics Committee of the Health Sciences Faculty of the University of Cape Town (IRB 00001938; REF 098/2004). The study was carried out in accordance with the criteria of the Declaration of Helsinki. Informed consent was obtained in writing from all participants and all information that might allow individuals to be identified has been deleted so as to protect their anonymity.

## Results

By addressing the question: 'What extra value did the PALSA PLUS *training *add?', we hope to shed light on those specific aspects of the PALSA PLUS training which may have contributed to the more effective implementation and subsequent use of the treatment guidelines in intervention clinics. We argue below that the added value of PALSA PLUS training included the use of an educational outreach training approach which facilitated interactive learning, thereby allowing for the integration of learning and practice and the provision of both emotional *and *operational support. By "operational" support we refer to the active facilitation of the management support required to effect all those infrastructural changes required to make implementation of new diagnostic and treatment options, as outlined in the guidelines, feasible and practical. In addition, the involvement of all clinic nurses (rather than ART nurses only) in the training appeared to facilitate the horizontal system-wide integration of HIV/AIDS care.

### Training as facilitated interactive learning

Nurses who had attended provincial training prior to PALSA PLUS training (i.e. ART nurses only) argued that although PALSA PLUS training covered much of the same material as the provincial training, it was more interactive and felt more like "training" and less like "an information session":

*"I went to the provincial training before we started PALSA PLUS. Ja [yes], uhm, it sounded OK but it was, to me, uhm, more superficial. ...I found when you wanted to ask some questions, you felt you needed a session for us at clinic level. ... It [provincial training] was a high quality lecture, it was sort of an information session. And when I went to PALSA PLUS then I felt, "****Now***, *we are being *trained." *(ART nurse 1, intervention site)*.

It was suggested that the PALSA PLUS training, which was conducted by HIV/AIDS and TB co-coordinators and designed specifically for nurses at clinic level, was less intimidating, more appropriate, and better attuned to the realities of primary care level treatment and care:

*"I felt intimidated [at the provincial training]. When you are a primary care nurse and you come with actual problems at grassroots and when you pose such questions among high-ranking academics – because we had people from universities and doctors from hospitals lecturing – so now, we are talking another language." (ART nurse 7, intervention site)*.

Nurses also valued the way in which the transfer of counselling skills was built into the PALSA PLUS nurse-training model and were keen to model the facilitative communication skills demonstrated by nurse-trainers in the course of their own patient encounters. In the following data extract, the nurse explains that, unlike the provincial training, the PALSA PLUS training demonstrated a more patient-centered approach to the promotion of drug-adherence:

*"The modules from province is like a lecture. How can I give a sick person a lecture? A frustrated, miserable person a lecture? We were going up too high above the patient, when it must be done more practically. And the patient should be more involved." (ART nurse 6, intervention site)*.

### Integrating learning and practice

Participants saw the PALSA PLUS on-site training approach, spread out over three to four months, as facilitating the gradual integration of new clinical information and skills. The integration of training and practical clinical work also allowed for ongoing supervisory feedback from nurse-trainers. This was seen to increase both the usefulness and the uptake of the training:

*"We had time to go and revise... It was one chapter a week and from one Tuesday to the next Tuesday you've seen many TB cases, you know, you've actually applied what you learnt. So it was actually better, the PALSA PLUS." (ART nurse 7, intervention site)*.

*"It's more relevant here [on-site] than outside because you can put it into practice. Whereas with off-site training, there's no evaluation of whether we are on the right track." (ART nurse 1, intervention site)*.

Participants nonetheless argued that their concentration and/or attendance at on-site training had suffered because of overlapping work-related commitments, especially the pressure of patients awaiting consultations. By comparison, centralized off-site training was seen to allow for more dedicated training time (i.e. a one-week block as compared to two-hour sessions over three to four months). Despite acknowledging the advantages of on-site training, some nurses therefore expressed a preference for training programmes which took them out of a stressful work environment. However, this disadvantage of on-site outreach training – the pressure experienced by nurses with patients awaiting their attention – is the flipside of what makes on-site training potentially more effective, through its immediate and direct link with 'real-life' patient service provision.

### Ongoing on-site training provides emotional support

Nurses' need for emotional support was raised frequently. In their interviews, all nurses emphasized the high emotional toll of their ever-increasing workload with HIV positive patients who, at this early stage of the roll-out, are generally very ill:

"If you are a human being, you will be touched [by the work with HIV positive patients]. It will work on your mind. You need to be strong and also develop skills to cope. Although, even so, you can't cope with everything." (ART nurse 6, intervention clinic)

Respondents noted that the ongoing engagement over time between PALSA PLUS nurse-trainers and clinic nurses provided them with much-needed support:

*"I found them [PALSA PLUS trainers] accepting us. ... They try to reassure us, 'No, we are there, whatever the trouble, you must phone me"'. (Generalist nurse 3, intervention site)*.

By comparison, respondents argued that little formal provision had been made at provincial level for providing support to nurses involved in the initiation of the ART roll-out. Inadequate support from clinic-level managers (referred to below as 'supervisors') was also commonly reported:

*"Our supervisors don't support us. They never come and ask, 'How are you, Can I help you?"' (ART nurse 2, intervention site)*.

Nurse-trainers also identified the supportive aspects of the PALSA PLUS training as one of its key successes:

*"No, the responses [to the training] are very, very good. They have a feeling of support". (Nurse-trainer 2)*.

The ongoing nature of the training over time was, in and of itself, a source of reassurance and support:

*"The trainers are really good. We ask them a question, and if they don't know, then the next week, they will come back with an answer." (ART nurse 2, intervention site)*.

Our observations of clinic training showed that the nurse-trainers' roles expanded from training in guideline use alone to include both the provision of emotional support and ART programme facilitation. For example, some nurse-trainers involved themselves in supportive system-level interventions, including liaising and negotiating for required operational changes with managers at various levels of the system. Some nurse-trainers also became actively involved in 'head-hunting' staff for vacant clinic ART posts and ensuring adequate ART supplies. This was possible because, as provincial-level HIV and TB co-coordinators, trainers were in a position to effect such interventions. The incorporation of prayer into nurse-training sessions, as a means of accessing spiritual reserves for emotional support, is an example of PALSA PLUS nurse-trainers incorporation of their own culturally appropriate support activities for nurses working at the frontlines of the epidemic [15]. The practical support and ongoing engagement on the part of trainers was especially appreciated by clinic nurses and may go a long way to explain the effects of the PALSA PLUS training and any differences in successful uptake of the guidelines between the intervention and control clinics.

### Horizontal system-wide integration of comprehensive HIV/AIDS care

Nurses all agreed that specialized ART nurses are needed within the PHC system for the vertical management, monitoring and counselling of ART patients. This, they suggested, would help to ensure treatment continuity and a stronger patient-provider relationship. However, nurses also noted consistently that all PHC nurses needed training in ART provision to better manage ART patients suffering from opportunistic infections or drug side-effects. Likewise, it was suggested that ART nurses needed to locate ART provision within a more comprehensive approach to AIDS care.

It is interesting to note that the elaboration of *non-ART related *AIDS care within PALSA PLUS was often especially valued by nurses who received the PALSA PLUS training. By comparison, nurses at control sites expressed a need for additional training, in both ART and non-ART related aspects of AIDS care, especially TB care:

*"The guidelines are simple and straightforward. But you do have to be trained to understand them, especially if you don't have PHC training, and especially if you don't know TB, which can be tricky if you [the patient] have HIV." (ART nurse 4, control site)*.

As mentioned previously, at control sites only those nurses designated as 'ART' nurses attended the provincial ART training and received their own copies of the PALSA PLUS guidelines. However, some of the PHC, or generalist, nurses at control sites made copies of their ART nurse colleagues' guidelines or specifically requested their own copies from provincial management. The guidelines were therefore widely disseminated – in the first year the document was printed three times to keep up with demand.

A PHC nurse at a control clinic (who had photocopied her ART colleague's PALSA PLUS guidelines) pointed out that the guidelines were especially valuable because they did not apply to ART provision or AIDS-defining conditions only, but to a wide range of conditions potentially exacerbated by HIV/AIDS:

*"The guidelines are like steps to follow. With regard to chest problems which are not responsive to antibiotics, it's guiding me properly – what steps to follow. Ja [yes], and it's not only that. There are so many conditions that they have put in." (Generalist nurse 9, control site)*.

By involving all clinic sisters in the training, PALSA PLUS presupposes that the treatment of ART patients should not be unnecessarily verticalized within the primary care context. In contrast, nurses at control sites suggested that, in the absence of training for PHC nurses, HIV/AIDS care as a whole had become specialized:

*"It is best if everyone gets the training [in ART]. We are encountering problems. It's as if ART is a speciality but these patients are everywhere, at [the PHC side] as well. HIV, ARV, TB: they go hand-in-hand." (ART nurse 10, control site)*.

*"The ART nurse shouldn't have to treat patients for minor ailments just because they are [HIV] positive. The ART side is full. If they [PHC nurses] could have undergone training they could have known how to treat an HIV positive patient..." (PHC nurse 9, control site)*.

PHC nurses at control clinics felt particularly strongly about their need for training in AIDS care, including ART provision. They argued that they needed to be better equipped to treat the PHC needs of AIDS patients and so refer only ART-related complications to ART nurses. It was pointed out that a vertical HIV/AIDS programme was not feasible as the numbers of patients on ART was increasing and burn-out amongst ART nurse was inevitable:

"Everything concerning HIV happens at the ARV site. But they [ART nurses] need relief. ... The nurses become exhausted. They become depressed."(PHC nurse 9, control site.)

*"At the end of the day, 90% of our patients [clinic-wide] are probably HIV positive." (PHC nurse 8, intervention site)*.

### Incorporating counselling skills into the PALSA PLUS model

Skills development in counselling, including adherence counselling, was incorporated into training and support workshops for nurse-trainers. However, both participant observation and interview data suggest that additional training may be required if nurse-trainers are to be effectively empowered to convey practical counselling skills in a consistent and appropriate way to clinic-based nurses. Participant observation of on-site training showed that nurse-trainers were quick to adopt a model of PHC nursing as holistic care-giving. However, many had difficulty in conveying the practical skills required for practicing a more patient-centered and less technical model of patient care. Interview data also suggest that many nurses, including ART nurses, continue to have a limited understanding of their counselling role. They tend to see this as the provision of advice regarding drug adherence and other health-related behaviours rather than as patient empowerment for informed decision making:

"Some of them [some patients]...You give advice, they jump and do it. But the others are so difficult." (ART nurse 2, intervention site)

In this extract, the nurse conceives of counselling as 'advice' that must be complied with, rather than as a process of supporting the patient's expressed needs. Some PHC nurses also reported that they insist on HIV testing *prior *to treating the presenting condition of patients in order to ensure that patients with potentially AIDS-related symptoms 'agree' to VCT (voluntary testing and counselling):

*"Sometimes, if you give them treatment, they'll not come back [for VCT]. ...I sit down and explain and explain until I win and then I go and hand them into the VCT counsellor's hands. I'm saying, 'See this lady and send her back to me so that she can have an appointment'. Because sometimes if you give them treatment, they'll not come back [for VCT]." (PHC nurse 8, intervention site)*.

Likewise, in order to achieve condom use or drug compliance, nurses stated that they resorted to a variety of persuasive strategies that are, arguably, at odds with a counselling role:

*"I threaten them. 'I'm going to go to the police and tell them you're not using a condom and that you are killing people'...." (ART nurse 7, intervention site)*.

Despite expressing these somewhat prescriptive and seemingly punitive views, there was nonetheless also a strong shared sense between nurse-trainers and nurses of their role as caring and compassionate community members committed to helping those with HIV/AIDS. This provided a framework for increased investment in counselling training:

*"I think emotional support and counselling must be, actually, in the [PALSA PLUS] guidelines because, after all, especially with these HIV people, they need that." (PHC nurse 8, intervention site)*.

The PALSA PLUS guidelines and training were designed principally to facilitate evidence-based syndromic care and cannot substitute for skills-training in counselling. However, as a result of these research findings, key messages regarding counselling were subsequently incorporated into an updated version of the guidelines and training. This was on the grounds that nurses in the Free State saw the PALSA PLUS guideline as the anchor of the ART programme. This was especially so given that, in the absence of any other standardized national or provincial guidelines at the time, the PALSA PLUS guideline functioned as a very powerful and practical manifestation of the Free State's ART programme.

## Discussion

The growing burden of HIV/AIDS in sub-Saharan Africa, and the urgent need to rollout treatment programmes, requires innovative approaches to building capacity for ART delivery and integrating comprehensive HIV/AIDS care within primary care. As ART becomes available more widely in many low and middle income country settings, it is becoming increasingly clear that human resources for health are the major constraint to ensuring that all those who need ART receive it, as part of a comprehensive package of care [31,32]. Little attention has yet been paid to how best to build capacity for ART rollout in under-resourced settings without depleting other components of the health service of scarce human resources. The PALSA PLUS intervention begins to address this by training all nurses – the largest cadre of health care professionals in South Africa and many other African countries – in the clinical management of HIV/AIDS and respiratory diseases.

The PALSA PLUS model of clinic-based educational outreach targeting all professional nursing staff is a significant departure from current training approaches within the Free State health services and South Africa at large, as well as many other low and middle income countries. These training programmes tend to rely on either distance education or intensive off-site training with limited staff coverage [33]. This research indicates that clinic-based educational outreach can result not only in training coverage for a higher number of health workers but may also contribute to PHC integration by providing training to all PHC nurses, thereby facilitating horizontal rather than vertical HIV/AIDS care. PALSA PLUS may therefore contribute to much needed efforts to integrate the treatment of HIV/AIDS into the public PHC system [32].

An additional advantage of the PALSA PLUS training approach is its duration over time, allowing the provision of initial supervision to nurses newly involved in ART delivery. Diagnostic and treatment difficulties can be brought back to the nurse-trainers' attention and learning can be immediately integrated and translated into practice. Regular contact between trainers and trainees is also advantageous in that it allows training to provide much needed emotional and psychological support for nurses bearing the brunt of the AIDS epidemic. This has been shown to be an important need in this and other settings [15,34,35]. The choice of district-based HIV/AIDS and TB co-coordinators as nurse-trainers placed these trainers in an especially strong position to bring emerging issues to the attention of district managers, as well as to engage both clinic and district-level management in resolving operational bottlenecks in the health system. In doing so, nurse-trainers were able to provide an additional level of support to clinic nurses.

Our findings indicate that contextual factors at play at this moment in the history of the Free State province's health-services ART roll-out may have resulted in a higher uptake of guidelines in control sites than can usually be expected of guidelines which are passively disseminated. These contextual factors include: (a) enthusiasm for the newly commenced ART roll-out and (b) the dearth of any other provincial guidelines regarding or including ART. Factors such as these may have made nurse enthusiasm for the guidelines as high in control sites (where guidelines were disseminated to ART nurses only) as in intervention sites (at which not just dissemination but training in guideline use for all nurses occurred). Any findings indicating improved treatment outcomes resulting from more effective guideline implementation at intervention clinics should arguably, therefore, be attributed in large part to the effectiveness of the training in facilitating guideline implementation, rather than simply to the relative failure of nurses at control clinics to engage enthusiastically with the guidelines simply because they were passively disseminated.

The PALSA PLUS training was thus perceived as going a long way in promoting, not just behaviour change at an individual health-provider level, but also a variety of broader system-level changes required to facilitate such individual behaviour change. A number of urgent health system challenges nonetheless remain which nurse-training alone cannot hope to address. Firstly, ART provision is more likely to be effective within a health system framework which consistently provides not only ART, but also a comprehensive package of PHC services ensuring prophylactic and curative treatment for opportunistic infections. Secondly, while a nurse-driven ART programme may help to address the problems resulting from an acute shortage of doctors, the fact remains that nurses at clinic level are also over-stretched, and that many PHC clinics are chronically under-staffed. This problem was expressed cogently by one of our respondents, *"I think that the biggest problem is going to be human resources at the clinic. I mean, we are going to run out of staff. I just see the ARV roll-out just getting bigger and bigger until a point. ... It's just going to become a huge problem." (Dr 1, Bloemfontein National Hospital)*. These are challenges not only for the Free State province of South Africa but also for health systems in other countries in sub-Saharan Africa [8,32,36]. Most of these countries are experiencing both critical absolute shortages in health care providers and poor distribution of providers within countries, exacerbated by deficiencies in skill mixes and poor physical and managerial infrastructures [8]. Addressing these deficits is key to improving health in Africa and reaching the Millennium Development Goals [37,38].

Findings suggest that training may have been insufficient to effect a deeper understanding of PHC providers' counseling role and that much more work needs to be undertaken in this area. Although counselling skills training was incorporated into the training intervention at the time of study, this was necessarily to a limited degree given the constraints on the programme. As a result of the findings of this study, relevant messages regarding the need for patient empowerment in decision-making have been integrated increasingly into the key messages elaborated in the guideline. The fact remains that effecting measurable improvements in those skills required to genuinely empower patients in decision-making may not be a realistic objective of the PALSA PLUS training intervention for some time. The PALSA PLUS programme necessarily prioritises its training objectives in order to achieve the best possible improvements in clinical treatment outcomes. What the programme currently aims to convey successfully is therefore not so much all necessary practical counseling skills per se, as an increasing conceptual appreciation of the rights of patients and of the roles of healthcare providers in helping patients towards autonomous decision-making on the basis of a neutral elaboration of their choices [39].

This evaluation of the PALSA PLUS training has several limitations. Firstly, its findings may not necessarily be generalisable to other settings in South Africa or beyond. This, however, is true for any programme evaluation, in so far as 'programmes differ from place to place because places differ' [40]. It nonetheless seems likely that the key aspects of the PALSA PLUS training programme identified by participants and outlined here are necessary if not sufficient to ensuring health practitioner behaviour change. Another significant limitation was that our study was limited to nurse perceptions and did not attempt to observe differences in nursing practices following training. The real effects of the PALSA PLUS intervention on nursing practices is considered by the RCT, the findings of which will be published separately. This process evaluation nonetheless shows that on-site training based on a 'cascade' model; drawing on the principles of educational outreach and interactive learning; and providing ongoing support to healthcare providers, is valued highly by primary care nurses as facilitative of their work. Of particular importance is ensuring that healthcare providers receive, not just the motivation, skills and knowledge to implement new diagnostic and treatment guidelines, but also the management support to effect all those system-level changes required to make guideline implementation feasible.

Finally, this evaluation did not address the sustainability of the training effects in the medium term. Ongoing efforts from district TB and HIV/AIDS co-coordinators and managers will be necessary to ensure that nurses continue to receive the support and ongoing training that they require in the context of an increasing demand for treatment within an already over-extended and under-resourced healthcare system [41].

## Conclusion

The incorporation of ART treatment into the PALSA guidelines and training appears to have provided the 'hook' with which to engage PHC nurses in a comprehensive approach to improving quality of care for a range of illnesses affecting both HIV positive and negative patients. The integration of AIDS care and ART provision into a comprehensive package of PHC is in line with strong calls from the South African AIDS research community to prioritise systems-based interventions on the ART funding agenda.

Nurses at both intervention and control sites valued the PALSA PLUS guidelines highly. The added benefits of the PALSA PLUS training were seen to include the training of all clinic nurses, thereby facilitating the clinic-wide integration of HIV/AIDS care and ART provision; the interactive learning approach; the provision of support; and the integration of learning and practice.

Despite these encouraging findings, there can be little doubt that nurse-training alone, no matter how well designed, cannot overcome the more fundamental problems facing publicly funded health systems in the Free State and many other African contexts. These include insufficient human resources and an increasing disease burden due to HIV and TB. As the roles and responsibilities of nurses are expanded, not only in ART provision but also in the overall delivery of care in the PHC system, adequate training and support to all those responsible for ensuring programme implementation must continue to be provided. This includes not only PHC nurses themselves, but all those involved in the planning and management of the programme at all levels of the healthcare system. Without this ongoing investment, we will not be able to protect what wealth we do have: the commitment and capacity of nurses to deliver comprehensive PHC, including ART.

## Competing interests

The authors declare that they have no competing interests.

## Authors' contributions

JS participated in the coordination and design of the study, executed the fieldwork and data analysis and drafted the manuscript. SL and LF participated in the coordination and design of the study, the critical review of the study findings, and the drafting of the manuscript. MZ, LF and EB conceptualized and initiated the PALSA PLUS programme. LF, PM, RE and AB designed and implemented the PALSA PLUS training programme. All authors read, contributed to and approved the final manuscript.

## Pre-publication history

The pre-publication history for this paper can be accessed here:


